# Real-Time Adaptation of an Artificial Neural Network for Transfemoral Amputees Using a Powered Prosthesis

**DOI:** 10.1109/TBME.2021.3120616

**Published:** 2022-02-18

**Authors:** Richard B. Woodward, Ann M. Simon, Emily A. Seyforth, Levi J. Hargrove

**Affiliations:** Center for Bionic Medicine, the Shirley Ryan Ability Lab, Chicago, IL 60611 USA, and also with the Department of Physical Medicine and Rehabilitation, Northwestern University, Chicago, IL 60611 USA; Center for Bionic Medicine, the Shirley Ryan Ability Lab, USA, and also with the Department of Physical Medicine and Rehabilitation, Northwestern University Chicago, USA.; Center for Bionic Medicine, the Shirley Ryan Ability Lab, USA.; Center for Bionic Medicine, the Shirley Ryan Ability Lab, USA, the Department of Physical Medicine and Rehabilitation, Northwestern University, USA; Department of Biomedical Engineering, Northwestern University, USA.

**Keywords:** Adaptive algorithms, machine learning, prosthetics, real-time systems

## Abstract

**Objective:**

We evaluated a two-step method to improve control accuracy for a powered prosthetic leg using machine learning and adaptation, while reducing subject training time.

**Methods:**

First, information from three transfemoral amputees was grouped together, to create a baseline control system that was subsequently tested using data from a fourth subject (user-independent classification). Second, online adaptation was investigated, whereby the fourth subject’s data were used to improve the baseline control system in real-time. Results were compared for user-independent classification and for user-dependent classification (data collected from and tested in the same subject), with and without adaptation.

**Results:**

The combination of a user-independent classifier with real-time adaptation based on a unique individual’s data produced a classification error rate as low as 1.61% [0.15 standard error of the mean (SEM)] without requiring collection of initial training data from that individual. Training/testing using a subject’s own data (user-dependent classification), combined with adaptation, resulted in a classification error rate of 0.9% [0.12 SEM], which was not significantly different (P > 0.05) but required, on average, an additional 7.52 hours [0.92 SEM] of training sessions.

**Conclusion and Significance:**

We found that the combination of a user-independent dataset with adaptation resulted in error rates that were not significantly different from using a user-dependent dataset. Furthermore, this method eliminated the need for individual training sessions, saving many hours of data collection time.

## I. INTRODUCTION

IT HAS been estimated that there will be approximately 2.2 million amputees in the United States by 2020, and that number is expected to continue to rise [1]. Transfemoral amputees—individuals who have lost their limb due to vascular disease, trauma, congenital disorders, or cancer [1]–[3]—constitute a large portion of this group. Despite the potential benefits of a prosthesis, a large number of these individuals stop using their devices [4], [5]; one study estimates that 11–22% lower limb amputees abandon their prosthesis within one year, with transfemoral amputees being twice as likely to abandon their device as transtibial amputees [6]. Improved prosthetic leg options that both provide greater functionality and are reliable and straightforward to use may help to reduce device abandonment.

Powered prostheses produce positive net power for the user, potentially reducing the effort and metabolic cost of ambulation activities [7]–[9]. These devices can enable (or enhance) the user’s ability to perform more complex activities, such as stair ascent, and often have pre-programmed modes such as ‘level-ground walking’ and ‘ramp descent’. However, switching between these modes can be cumbersome for the user. Various methods have been introduced to avoid this problem, such as a manual compensatory movement (e.g., fast, exaggerated movements from the hip or knee to lock joints into place for stair ascent) [10], or the use of an external control device (e.g., a switch or smartphone application) [11]. However, these approaches are inconvenient and do not allow seamless, automatic transitions between ambulation modes.

Machine learning approaches have shown promising results for powered prosthesis control, both in offline analyses and in real-time applications [12]–[20]. Many methods make use of supervised learning, whereby sets of new observations are grouped into labelled categories. The wide range of available algorithms—from the simplistic and computationally efficient [15], to complex deep-learning methodologies [21]—are all subject to the same limitation: they require properly labelled data to train the classification model. User-dependent classification, whereby data is collected from a single user to train the algorithm, which is subsequently tested against that user, has been found to be the most accurate method [22]. However, this approach is also the most time consuming for the user. Data collection session(s) can last hours and spread across days, often requiring repeated actions in a controlled environment, overseen by a trained investigator. In previous studies, we have investigated using an algorithm trained using a grouped dataset, comprising data from numerous users, and then applied to a unique individual, which we refer to as user-independent classification [22]–[24]. Although this approach often produces less accurate results than the user-dependent model, it removes the need for data collection sessions from the individual subject, and thus is faster and less burdensome to implement.

The work presented here extends this idea, whereby a trained ‘baseline’ classifier built on a user-independent dataset is further improved, or ‘adapted,’ using an individual’s unique data, during real-time use. We compare user-dependent and user-independent classification methods and analyze the effects of adaptation. The objective of this study was to determine if real-time adaptation of a user-independent dataset provides a significant improvement in classification accuracy. Furthermore, we examine the benefits of user-independent classification with adaptation against user-dependent classification, taking into consideration time requirements and other ‘real-world’ considerations.

## II. METHODOLOGY

### A. Experimental Protocol

Four individuals (2 male (both K-level 3) and 2 female (one K-level 3, one K-level 4)) with unilateral transfemoral amputations (one left leg, three right leg), aged between 32 and 69 [53.25 mean, 7.98 SEM], time since amputation between 17.1 and 48.6 years [34.13 mean, 7.09 SEM], height between 165 and 180 cm [173 mean, 3.14 SEM], and weight between 66 and 86.2 kg [76.53 mean, 5.01 SEM] participated in this study, with approval from the Northwestern University Institutional Review Board. Written and verbal consent was obtained from each participant.

**Fig. 1.**
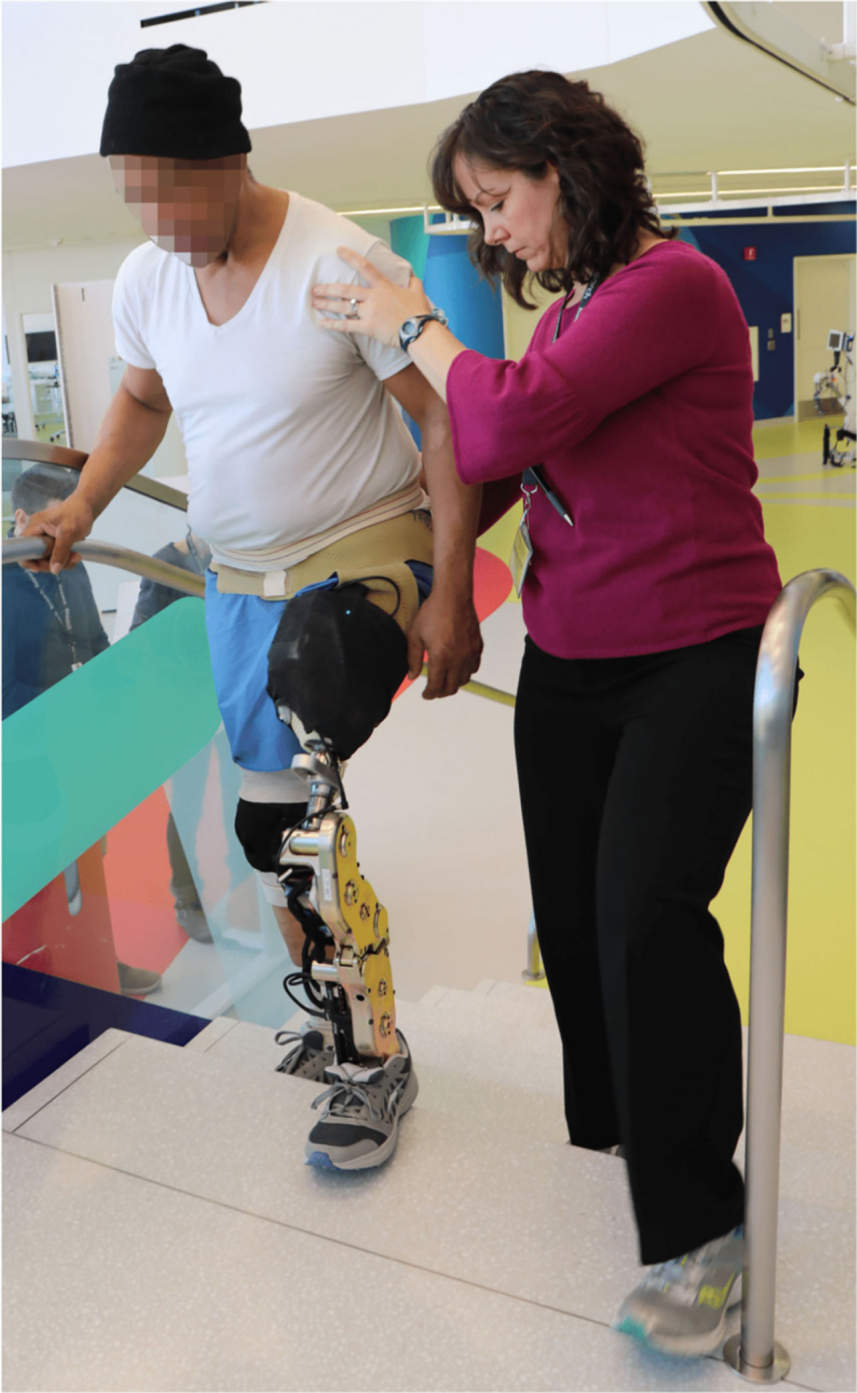
Participant using the second-generation powered knee/ankle prosthesis from Vanderbilt University.

All participants were fitted with a second-generation powered knee and ankle prosthesis developed at Vanderbilt University [25] (Fig. 1). The participants had previously used this leg and were familiar with its functionality. Prior to this study, the parameters for the powered prosthesis were configured for each subject using a method previously described in detail [26].

This study was conducted in a controlled laboratory environment. Participants were asked to attend training sessions of between 2–4 hours each day, for four days. For the remainder of this article, consecutive days will be referred to as consecutive sessions. A full break down of the times required for each session is shown in Table I. Each session involved several repeated tasks, including standing, walking, ascending and descending ramps, and ascending and descending stairs. Furthermore, each session included donning/doffing of the device and practice using the device, as well as unscheduled events such as fatigue (and need for breaks) or fitting/technical issues; these episodes caused corresponding variations in session lengths. This protocol has been described in detail in a previous study [27].

An experienced investigator used a wireless key-fob to transition the prosthesis between tasks (e.g., level-ground walking to stair ascent), and to label the activity that the user was performing. This was used as the ground truth for classification. Data from several mechanical sensors were also collected, including relative knee and ankle positions, knee/ankle velocities, and commanded joint torques. Furthermore, a one degree of freedom (DOF) load cell and a six DOF inertial measurement unit (IMU), mounted midway through the shank, were sampled. A complimentary filter was used to compute the angle of the thigh and the angle of the shank relative to vertical. Axial load information was used to segment the gait into stance and swing phases, and a corresponding trigger vector was produced as the state machine transitioned through states. All sensors were situated on the prostheses, with no additional sensors on the contralateral leg or the thigh. The electronics partially visible on the subject’s prosthetic socket in Fig. 1 are the embedded system and its battery.

### B. Signal Processing

The powered leg was controlled by a Linux-based embedded system with a Logic PD SOMDM3730 running at 600 MHz. Data were sampled at 500 Hz and were stored onboard a solid-state memory card. However, signal information—including state-machine transitions, which occurred at 30 *ms* frame increments—were streamed wirelessly to a laptop for real-time viewing and verification.

Offline data processing was performed using Python with the NumPy and SciPy packages. Data were normalized between subjects to account for side of amputation: for the subject with a left leg amputation, the appropriate mechanical channels were transformed (sign reversed) prior to processing. Online data processing was also performed using Python, albeit on the embedded system detailed above.

We processed the data in two stages. First, a ‘baseline’ classifier was created through offline analysis. In this stage, data from sessions 1–3 were organized, windowed, processed, and classified. This stage was performed using a personal computer, and the resulting classifier weights were saved for use in real-time processing, including adaptation. The second stage was a simulated online analysis that used the remaining data from session 4, and the previously saved classifier weights from the offline analysis. This stage processed data in two separate ways. First, the session 4 data were passed through the classifier using only feedforward estimation, and the collective results were reported as the control (i.e., no adaptation applied). The second method, ‘adaptation,’ attempted to improve the classifier and update the weights by classifying newly available data in real-time. Both stages are described in detail below. It is important to note that this study performed pseudo-online analysis, in that classification was performed in real-time on the embedded system, with real subject data from an existing dataset; however, the classifier estimate had no control influence on the powered prosthesis and therefore the classification occurred in an open-loop configuration.

### C. Feature Extraction

#### 1) Forward Prediction:

**Fig. 2.**
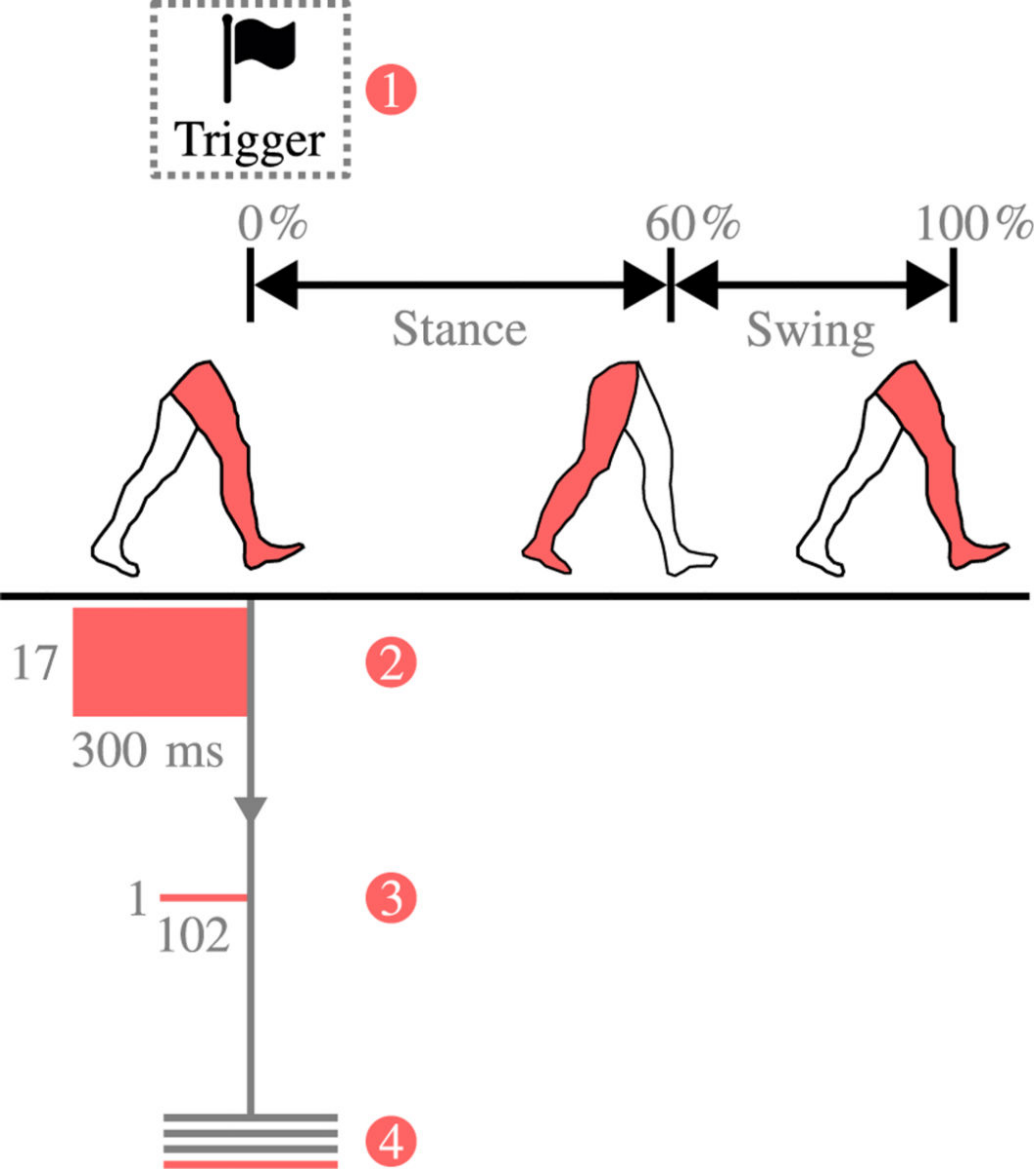
Visual presentation of feature extraction for forward prediction. (1) A trigger of interest is thrown. (2) A raw data window, comprising 300 *ms* of data (backwards, from the trigger point) and information from 17 mechanical sensors, is binned. (3) Features are extracted from the raw data window, resulting in a 1×102 feature vector. (4) Feature vectors are saved for future processing, labelled with a ground truth obtained from the trigger information.

Fig. 2 shows a visual representation of the feature extraction process for forward prediction, the process whereby data windows were used to estimate future gait activity, as intended by the user.

During key stages of gait, as the prosthesis transitioned through its state-machine, triggers of interest (TOI) were thrown that identified a pre-set list of gait events (Fig. 2(1)). From the TOI, working backwards, a 300 *ms* window of raw mechanical sensor data was binned (Fig. 2(2)). Six features were extracted from each sensor channel within this window, including: mean, standard deviation, min, max, initial value, and final value [15], [27]. These six features, multiplied by the 17 mechanical channels, produced a 1×102 feature vector per bin (Fig. 2(3)). Finally, these features were labelled with a ground-truth locomotion mode obtained from the trigger information and saved for classification (Fig. 2(4)). Labels were either standing (ST), level-ground walking (LW), stair ascent (SA), stair descent (SD), or ramp descent (RD). For these data, the prosthesis used the same impedance parameters for ramp ascent as level-ground walking [26], therefore all ramp ascent triggers were reassigned as level-ground walking. Furthermore, the data were labelled based on their step-type, to indicate if the step changed from one mode to another (transitional [T]) or remained in the same mode (steady-state [SS]).

#### 2) Backward Estimation:

**Fig. 3.**
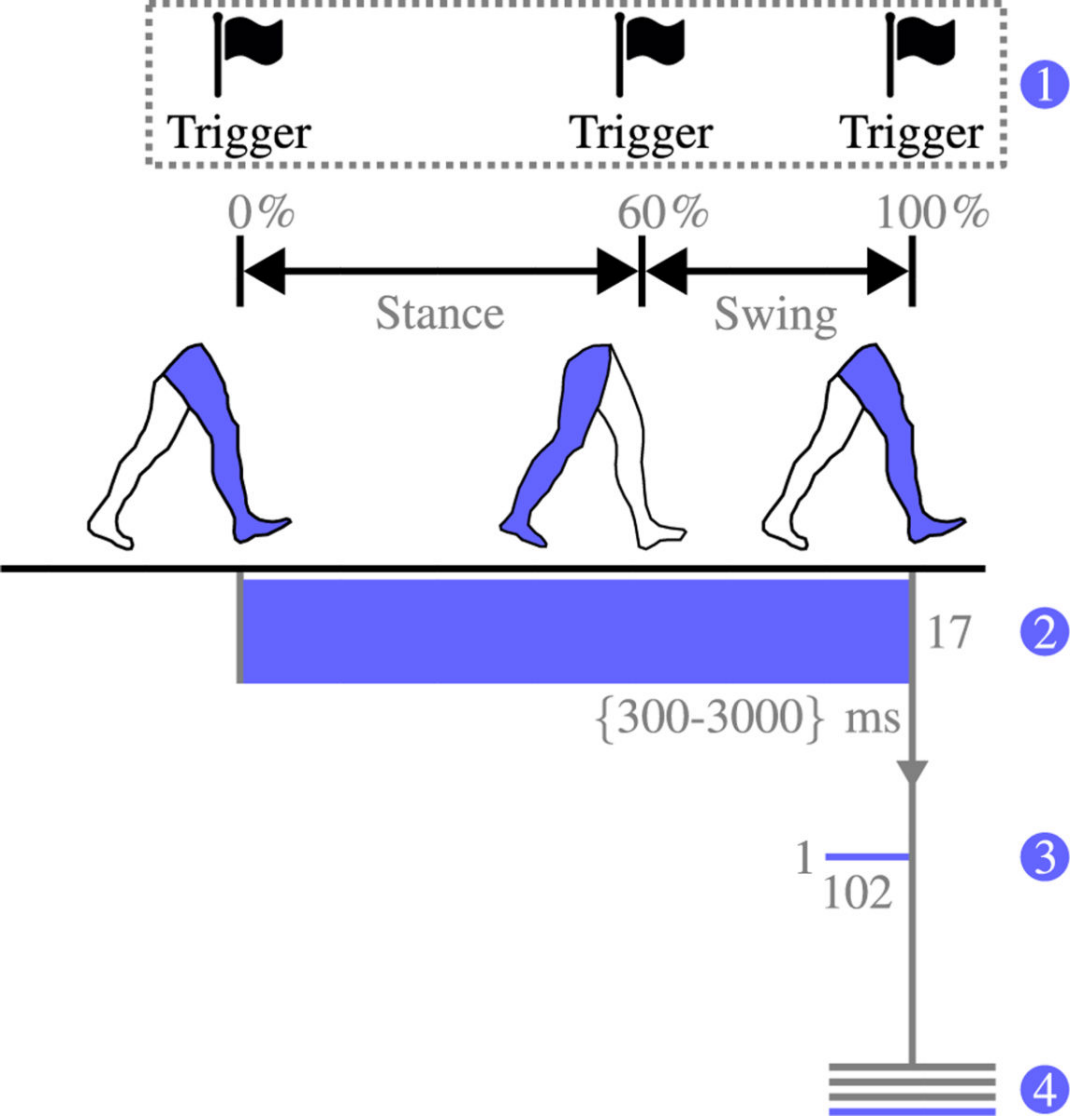
Visual presentation of feature extraction for backward estimation. (1) A sequence of triggers is thrown. (2) A raw data window, between the first and last trigger in the sequence, is binned. This window varied between 300 and 3000 *ms* and consisted of information from 17 mechanical sensors. (3) Features are extracted from the raw data window, resulting in a 1×102 feature vector. (4) Feature vectors are saved for future processing, labelled with a ground truth obtained from the trigger information.

To adapt the classifier in real-time, a technique called ‘backward estimation’ was used [24]. This technique extracted features from a larger data window during gait, which allowed for more accurate estimations of previous gait activity when classified. Although key-fob information was available, which influenced the state-machine that generated the trigger vectors used as ground-truth in our offline training, this data would not be available in a real-world scenario and therefore estimations during backward estimation were used as classifier targets in our pseudo-online study. Fig. 3 shows a visual representation of the feature extraction process for backward estimation.

Like feature extraction for forward prediction, trigger information was used to initiate the data windowing for backward estimation; however, for this technique, a group of sequential triggers were used, instead of just one (Fig. 3(1)). If these triggers, which spanned a complete stride (from heel-contact to heel-contact), were also determined to be a TOI, a data window, which varied in size between 300 and 3000 *ms* (larger and smaller windows were rejected), was binned (Fig. 3(2)). Despite the variable sizes of these raw data windows, the same features were extracted as for forward prediction, which resulted in the same 1×102 feature vector (Fig. 3(3)). Finally, these feature vectors were paired with a label for classification, which was obtained from the trigger information (Fig. 3(4)). These labels represented the same locomotion modes as described for forward prediction.

### D. Classification

#### 1) Offline Analysis:

Both the forward prediction and backward estimation feature windows were classified using a Scaled Conjugate Gradient (SCG) Artificial Neural Network (ANN) [28]. Previous work has shown that, compared to a linear discriminant analysis method, a SCG ANN has a lower estimation error and is more computationally efficient [23]. The SCG ANN had one hidden layer and 20 hidden neurons with a hyperbolic tangent activation function. Each classifier was trained over 1000 epochs with a break clause once the training error improvement plateaued at a precision of 0.001. Weights were initialized randomly between a range of:

(1) $$\left(\frac{-1}{\sqrt{fv}},\frac{1}{\sqrt{fv}}\right)$$

Where *fv* represents the number of inputs, which in this case is 102, resulting in initial weights between ±0.099. Through trial and error, it was found that there was no benefit from dimensionality reduction using principal component analysis, therefore this was not used.

Feature vectors were split into 85/15% datasets, for training/testing, respectively. The weights produced from the offline analysis were saved and used in our pseudo-real-time application. For brevity, the results of the offline analysis are not presented here.

#### 2) Online Analysis:

Estimation of locomotion modes was achieved by a feedforward neural network with the same structure as the SCG ANN detailed above. However, adaptation was performed using a gradient descent (GD) ANN with one hidden layer, 20 hidden neurons, a three-epoch break clause, and a learning rate of 0.01. The three-epoch break clause, found through trial-and-error, allowed for the best ratio of classification performance to computational speed on our embedded system. As the GD ANN had an identical layer/neuron hierarchy to the SCG ANN, this more simplified neural network could be used in lieu of the SCG for adaptation.

### E. Real-Time Adaptation

For the online analysis, data from session 4 were used in a pseudo-real-time test. To determine the effects of adaptation, data were processed both with and without adaptation.

**Fig. 4.**
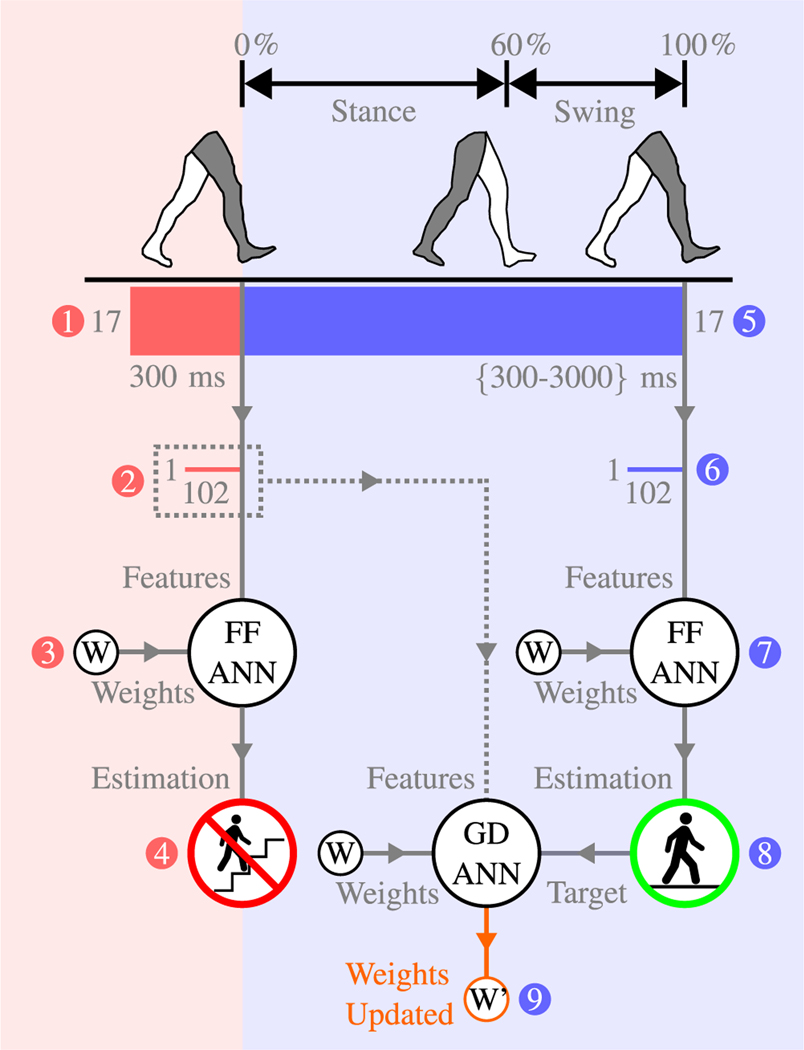
Visual presentation of the real-time evaluation. Forward Prediction: (1) A raw data window is binned, comprising 300 *ms* of data and information from 17 mechanical sensors. (2) Features are extracted from the raw data window and saved for future processing. (3) The saved feature vector, as well as the saved classifier weights from offline processing, are passed to a feedforward neural network (FF ANN). (4) The FF ANN estimates a locomotive mode. In the hypothetical example shown here, the algorithm has made an incorrect estimation of ‘stair ascent’. Backward Estimation: (5) Between two sequential heel strikes, another (larger) raw data window is binned, varying in size between 300 and 3000 *ms*. (6) Features are extracted from the larger raw data window. (7) The new feature vector is passed to an FF ANN, along with the same weights used in step 3. (8) Another (theoretically more accurate) estimation is made for locomotive mode. In this hypothetical example, the correct mode of ‘level-ground walking’ is estimated. (9) The feature vector from step 2 is trained, using the estimation from step 8 as the training target, and the same weights used in steps 3 and 7. This training process results in updated weights, which are used in future classification. Only steps 1–4 were run during data processing in the control methodology (forward prediction; red background), whereas all steps (1–9) were run in the adaptation methodology (forward prediction and backward estimation; red and blue backgrounds).

With no adaptation (control), the online analysis was simply a feed forward estimation, using the classifier weights saved during the offline analysis for classification. Fig. 4 shows a visual representation of control methodology in the forward prediction segment (red background)—data were windowed, features extracted, and features passed through an ANN for estimation (Fig. 4(1–3)). Estimation results (Fig. 4(4)) were saved, and their accuracy was compared against a ground truth which was logged by an investigator using a key-fob. In the hypothetical example in Fig. 4(4), the classifier has made an incorrect estimation of stair ascent, when the actual ambulation mode is level-ground walking.

In the adaptation methodology, the activity estimation classifier is reinforced in real-time through additional learning. Fig. 4 shows a visual representation of the adaptation methodology in both the forward prediction segment and backward estimation segment (blue background). In the same way as described for the control methodology, forward prediction data windows were formed, their features extracted, and an estimation predicted (Fig. 4(1–3)). However, for the adaptation methodology the feature windows were saved for future processing.

A backwards estimation window was formed between two sequential heel strikes (Fig. 4(5)). Features were extracted from these windows and passed through the ANN to obtain a more robust estimation (Fig. 4(6–8)). As previously mentioned, the broader data window in backward estimation allows for a more accurate classifier, and, in the example shown in Fig. 4(8), the ANN predicted the hypothetical correct activity: level-ground walking.

Adaptation was performed using a GD ANN and three key in put components : the saved features during the forward prediction stage as the input data (Fig. 4(2)), the activity estimation from the backward estimation ANN as the training target (Fig. 4(8)), and the classifier weights. Following three training epochs, the GD ANN produced updated weights, as seen in Fig. 4(9). This cycle was repeated, and the updated weights used in future estimations to allow the classifier to adapt to new data.

It is important to note that the goal of the backwards estimation stage is to produce an accurate ground truth label, which the saved forward prediction features are trained against

The forward predictor and backward estimation have separate classifiers, and the 300–3000 *ms* backward estimation feature windows—swhich contain both swing and stance phase information—should not be directly compared to the forward predictor windows, which only contain swing phase information.

### F. Embedded System Real-Time Speed Test

To determine the suitability of running the full adaptation process with the GD ANN in real-time, computational processing time was recorded while classifying and adapting using the embedded system. This process was repeated three times, on three different tasks/activities with in the same collection session, including a file containing just stair activity, a file containing just level-ground walking activity, and a file containing a combination of gait types (a circuit), which included stair ascent/descent, walking, ramp ascent/descent, and standing.

The test was performed in three configurations:

- No classifier running: All system functions to control the powered leg, including data acquisition, state machine operations, and motor outputs (although not physically connected to motors).
- Classifier running; control mode: All system functions as above, with the classifier running, albeit only running the control methodology.
- Classifier running; adaptation mode: All system functions as above, with the classifier running, with adaptation processing enabled.

The average sample processing time for no classification should be approximately 30 *ms*, which is the length of each frame increment on our embedded system. The addition of classification with adaptation should not substantially increase that sample time, otherwise a computational bottleneck would occur.

### G. Performance Evaluation

For each participant, sessions one to three were used as training data, whereas session four was used in testing. The dataset Blocks were created by sequentially grouping sessions:

- Block^1^: Information from session one.
- Block^2^: Information from sessions one and two.
- Block^3^: Information from sessions one, two, and three.

**Fig. 5.**
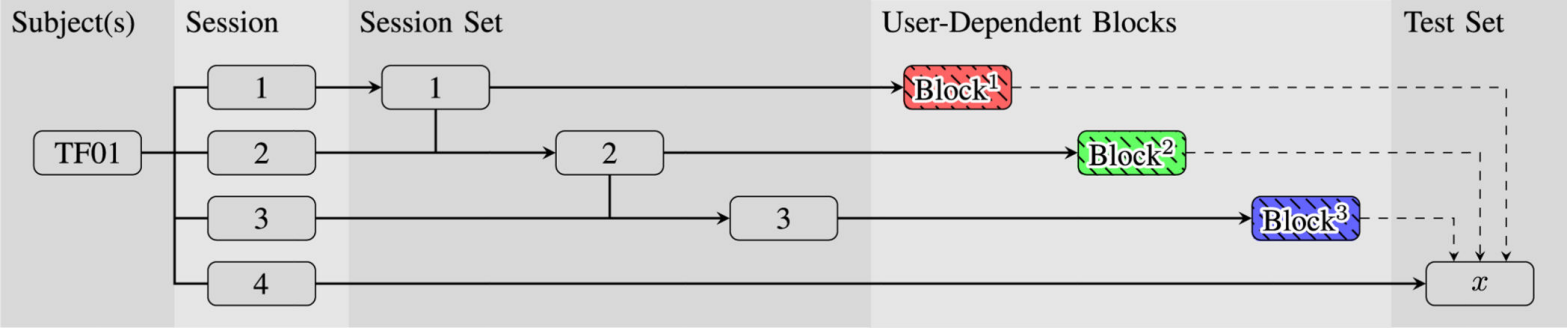
Example of the training/testing permutations when classifying in User-Dependent Classification (DC) mode. The figure shows an example of creating user-dependent dataset blocks from a single subject to be tested against the same subject.

**Fig. 6.**
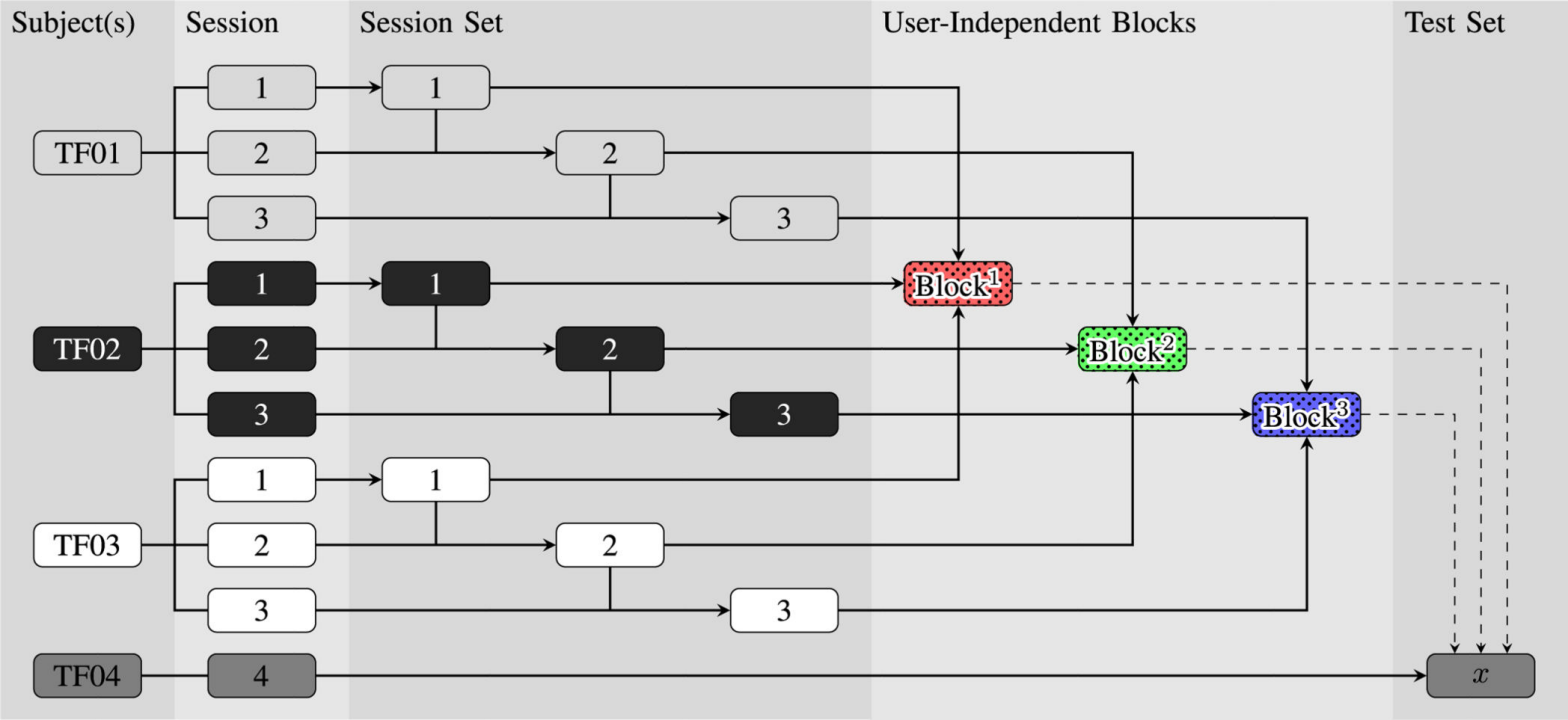
Example of the training/testing permutations when classifying in User-Independent Classification (IC) mode. The figure shows an example of grouping data from three subjects to create user-independent dataset blocks, to be tested with data from a unique fourth subject.

These Blocks were processed using two classification methods that have been used previously by our group [22]:

- User-Dependent Classification: Classifier was trained and tested using the subject’s own data. Training data were grouped by session, creating three dataset Blocks. This method is shown in Fig. 5.
- User-Independent Classification: Data were grouped between participants to create dataset Blocks. Like the User Dependent Classification mode, data were first grouped by session for each participant, before being grouped across participants using a leave-one-out method. Three dataset Blocks were created from three subjects and were tested against data from a fourth subject. This method is shown in Fig. 6.

Classification error rates for all blocks, groups, and control/adaptation methodologies were determined using a ground truth, obtained by an investigator using a key-fob. Furthermore, classification processing times on the embedded system were compared to establish whether this proposed adaptation methodology impedes real-time performance.

### H. Statistics

Statistical analysis was performed in Minitab (version 18.1), using a mixed effects model with population subject identifier as a random factor, and session blocks(Block^1^,Block^2^, Block^3^), data processing type (user-dependent classification or user-independent classification), and adaptation type (no adaptation [control] or adaptation), as fixed factors. The model was performed with 3^*rd*^ order interactions, restricted maximum likelihood variance estimation, and a Kenward-Roger approximation test method for fixed effects. Pairwise comparisons were performed using a Bonferroni (95% confidence level) method.

## III. RESULTS

**Fig. 7.**
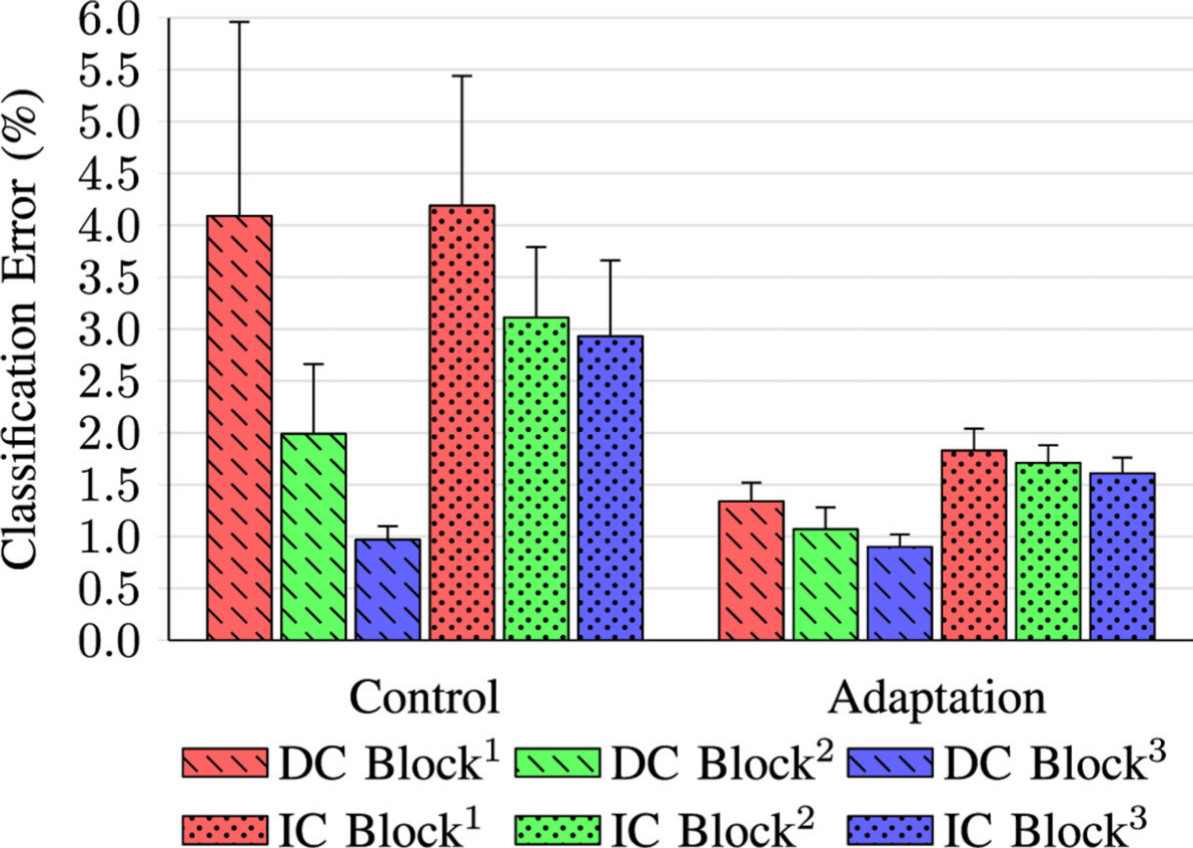
Bar graph showing the results from all dataset groups, including subject grouping (user-dependent classification (DC) and user-independent classification (IC)), session blocks (Block^1^, Block^2^, and Block^3^), and without adaptation (control) or with adaptation. Error bars are the standard error of the mean (SEM).

Table II and Fig. 7 shows the results from all dataset groups, including subject grouping (user-dependent classification and user-independent classification), session blocks (Block^1^, Block^2^, and Block^3^), and with or without adaptation. Results are presented as the collective classification error (%) and standard error of the mean (SEM), while Table II also separates results into steady-state and transitional errors, as well as the combined weighted average. Confusion matrices showing the classification error rates across locomotion modes can be found in Table III.

Offline classification required 16.65, 38.29, and 57.62 minutes to process data from all four subject for block configurations 1, 2, and 3, respectively (Intel i5 3570 K, 8 GB RAM, AMD RX 480). Computational processing times on the embedded system for ‘no classifier running,’ ‘classifier running; control mode,’ and ‘classifier running; adaptation mode,’ all had an average sample run time of 30 *ms* ± 50 *μs*. Nominal real-time classifier processing times were 1.69 and 3.66 *ms*, for ‘classifier running; control mode’ and ‘classifier running; adaptation mode’ mode, respectively.

Statistical results are shown in Table IV, with a Standard Error of the Estimate of 0.015 and an R^2^ of 26.84%. The fixed effects of Blocks (*P* < 0.05) and adaptation (*P* < 0.01) were found to be significant, however, none of the interactions or other fixed effects were found to have statistically significant effects (*P* > 0.05).

## IV. DISCUSSION

For both classification models (user-dependent or user-independent) and adaptation types (control or adaptation), addition of more training data improved classifier accuracy (as shown by differences between Block groups in Fig. 7), with lower classification error rates and decreased SEM.

The user-dependent control group showed the biggest improvements, for both error rate and SEM, with consecutive Block groups. In Block^1^, with a training to testing dataset size ratio of 1:1, any physiological change or difference in gait performance between sessions impacted testing performance far greater than in Block^2^ (2:1 training to test ratio) or Block^3^ (3:1 training to test ratio), therefore a larger training dataset containing more varied data across different sessions is advantageous.

Applying adaptation to user-dependent classification resulted in a noticeable decrease in classification error and SEM. Although an improvement was also seen as session information was increased between Block groups, a single session of training data (Block^1^) resulted in lower classification error rates than those from Block^2^ of the user-dependent control group.

The user-independent control group also saw improvements in both error rate and SEM as training data were increased across Blocks. However, due to increased variability in the data—likely the result of each subject’s unique gait characteristics—the improvements in classification accuracy from Block^1^ to Block^3^ were less pronounced than in the user-dependent classification control model, with a corresponding reduced improvement in SEM. Likewise, although adaptation reduced classification error rates and SEM across each Block, the improvement between Blocks was limited in the user-independent classification model, perhaps because a saturation point was reached, which restricted further improvement of the classifier.

Error rates were expectedly higher in transitional steps, when compared to steady-state. Due to known additional complexities in the transition kinematics [29] and because far fewer transition steps were available for classification, transitional estimation errors were often a magnitude higher than steady-state, as seen in Table II. However, because transitional steps comprise only a sub-set of the entire dataset, their higher error rates have a minimal impact on the collective average error.

Error rates were also expectedly higher in locomotive modes which occurred less frequently, such as the stairs and ramp modes. While standing (ST, 38%) and level-ground walking (LW, 46%) made up the majority of the total gait activity, there was far less training data available for stairs ascent (SA, 5%), stairs descent (SD, 7%), and ramp descent (RD, 5%). Because of this, SA/SD/RD often had higher errors than ST/LW, as seen in Table III. However, they also typically showed greater improvements through an increase in training data (Blocks) and with adaptation applied.

Collectively, these results show that the best scenario was to train, test, and adapt using the subjects’ own data (user-dependent classification), which resulted in a 0.9% [0.12 SEM] error rate (with three days of training data), although this requires the user to provide all the necessary training data themselves. Alternatively, using a user-independent classification model with adaptation resulted in a comparable1.61%[0.15SEM] error rate (with three days of training data), while also potentially saving hours of individual training time. User-independent classification Block^1^ with adaptation produced lower classification and SEM errors than user-dependent classification Block^2^ without adaptation. Furthermore, our results show that one session of data collection with adaptation can outperform two sessions of data collection with no adaptation, which could greatly improve intent recognition with limited datasets. These results suggest that training a classifier after a single collection session, then adapting it during a second online collection session, could reduce error rates by 0.65% and 1.28% for user-dependent classification and user-independent classification, respectively.

While certain expectations can be made from individual subject performance in the user-dependent classification model, due to the similarity of training and testing datasets, the performance of a user-independent classification model is largely reliant on the individuals within the dataset. While a user-independent dataset consisting of individuals with similar physiological characteristics is likely to work well for other users with comparable characteristics, this same dataset may not work well for users with vastly different attributes. Although a varied user-independent dataset is not likely to be a perfect match for any individual subject, it is possible to create a suitably good ‘baseline classifier’ for a wider array of individuals, which we believe is the best course of action when combined with the novel adaptation method described here. In theory, a large enough dataset from enough subjects should work for any unique subject, regardless of their characteristics. However, the optimal number of subjects and variety of subject characteristics necessary to create such a dataset is currently unknown.

Although difficult to compare across studies, due to variability in algorithms used, numbers of subjects, and locomotive modes identified, the results presented here are similar to those reported in recent literature. Liu *et al.* used both mechanical channel information and electromyography in their online adaptation classification strategy. Their study recommended an entropy-based adaptation method for a real-time human-in-the-loop study, showing a classification error as low as 4.80% [30]. Bhakta *et al.* used the XGBoost algorithm and split their analysis into user-dependent and user-independent subject grouping. They reported classification error rates of 3.81% and 10.11%, respectively, although their study did not include online analysis [31].

When compared against prior results from our own group, similarities and improvements can be seen. In earlier work (n=6, same powered prosthesis, same ANN, single collection session, user-dependent and user-independent classification methodology, offline analysis), we reported an average error of 1.12% and 3.25% for user-dependent and user-independent classification, respectively, with no adaptation [23]. Although reporting a higher classification error rate of 4.03%, Spanias *et al.* (n=8, same powered prosthesis, LDA classifier, two collection sessions, user-dependent classification methodology, online analysis) used a ‘true’ online study, which adds an additional level of complexity, although it is more realistic than the present study [32]. Young *et al.* (n=8, same powered prosthesis, dynamic Bayesian Network classifier, one collection session, user-dependent and user-independent classification methodology, offline analysis) showed a steady decrease in classification error from 18% and 12% as the number of subjects included in the user-independent dataset increased from one to seven, respectively [22]. Simon *et al.* (n=2, same powered prosthesis, ULDA classifier, up to four collection sessions, user-dependent classification methodology) reported improved classification errors when increasing the number of training sessions for intent recognition [33], which is comparable to that seen in the present study.

Although individually the fixed effects of adaptation (*P* < 0.01) and Blocks (*P* < 0.05) were statistically significant, no interactions between the groups shown in Fig. 7 were found to be statistically significant. The results of the mixed effect model were compelling, because while the fixed factor terms of adaptation and Blocks do significantly affect the response, the other factors (including 2^*nd*^ and 3^*rd*^ order interactions) did not. This is especially important for the ‘Classification’ fixed term, as that would suggest that there was no significant difference between user-dependent and user-independent processing types, and could indicate a benefit in grouping user data, regardless of additional adaptation.

This study showed that combining a user-independent classification model with individualized adaptation had the biggest reduction in participation time. Each collection session in this study varied between two and four hours (Table I), and Block^3^, which contained three sessions of training data, averaged a total of 7.52 [0.92 SEM] hours of data collection across all subjects.

A unique subject, using the powered prosthesis for the first time, with no prior training sessions, could expect a suitable baseline classification error rate of 2.93% (Control Group, IC Block^3^ - Fig. 7), before adaptation. With adaptation, over a single session (approximately two hours), this error rate could reduce further to 1.61% (Adaptation Group, IC Block^3^ - Fig. 7). This error rate sits between those of Block^2^ and Block^3^ of the user-dependent classification control group, saving the user multiple hours of data collection sessions. Realistically, however, an individual with no prior experience with powered prostheses would have to overcome a large learning curve during their first use, which must be taken into consideration when applying these results to real-world scenarios. All subjects tested within this study had prior experience with this powered prosthesis.

Although this study demonstrated improvements in classification accuracy with up to three sessions of training data, it is unclear if more sessions would further improve results. Furthermore, the results seen here are based on data collected entirely in a controlled laboratory environment, and therefore it is possible that data collected in the home or other uncontrolled settings, with increased variability, may affect these findings. We speculate that a greater number of subjects would provide more robust results, however, it is also possible that a saturation point was reached, in that further improvements are limited by the current methodology.

The GD ANN was chosen for use in real-time adaptation due to the limited computational power on the embedded system, and the need to process adaptation within 30 *ms* sample windows. The GD method could perform much faster than the SCG method; a three-epoch loop of a GD ANN could be run comfortably given the hardware and time constraints. Results from the real-time analysis of the embedded system showed that the classifier performed efficiently. The difference between no classifier, and the classifier running with adaptation, was less than 50 *μs*, and therefore our GD ANN added no noticeable computational bottleneck and did not impact any operation of the powered prosthesis.

Although this study used a proprietary Linux-based embedded system, there are no foreseen reasons why real-time feature extraction, forward prediction, and adaptation could not be performed on alternative commercial systems, such as the Raspberry Pi or BeagleBone platforms - both of which are also capable of running a Linux operating system.

This study has some limitations. Although physiological characteristics (height, weight, gender) of subjects were not subjectively similar, they all exhibited similar activity levels (K3/K4 ambulators) and had similar amounts of experience with the powered prosthesis. Subject numbers were also quite low (n=4). Due to this study’s ‘pseudo’ real-time application, the classifier had no influence on the activity of the prosthetic limb. Conversely, the subject’s reaction to the change in gait, which is expected to impact performance in a true real-world scenario, is also not accounted for in our results. Another limitation is that the backward estimation was used as a ground truth in training, and this may not always be correct. Although the backward estimation is far more accurate than the forward prediction, it is not entirely error-less and could train new observations with an incorrect target.

The classification results presented here are comparable to those seen in recent publications, however, this study has further demonstrated that such results are potentially achievable with far fewer training requirements for unique users. This article has established a training/testing model that is easy to apply to new subjects, is computationally efficient to run on non-specialized embedded hardware, and is potentially both practical and feasible for use across diverse environments.

## V. CONCLUSION

This study shows that pairing a user-independent classification model with real-time adaptation results in comparable classification accuracies to user-dependent classification, but with a substantial reduction in individual training time.

With a reasonable baseline classification accuracy, we have demonstrated that adaptation using newly available data from a unique subject results in improvements in gait estimation over a short period of time in a single data collection session. This methodology has the potential to enable transfemoral amputees to use their powered prosthesis with fewer training sessions, without sacrificing safety or control accuracy.

We have also established that an ANN can comfortably operate and adapt new data in real-time on an embedded system; however, future work is necessary to apply this technique to real-world use and operation of a powered prosthesis.

## Figures and Tables

**TABLE I:** BREAKDOWN OF THE TME REQUIREMENTS (HOURS(H)MINUTES) FOR EACH PARTICIPANT IN THIS STUDY. SESSIONS ONE TO THREE WERE USED FOR TRAINING THE CLASSIFIER, SESSION FOUR WAS USED FOR REAL-TIME TESTING/ADAPTATION

Subject		01	02	03	04

	Session 1	2h30	2h00	2h45	2hl5
Training	Session 2	2h45	2h00	4h00	2hl5
	Session 3	2h20	2h00	3h20	lh55
	
	*Total*	*7h35*	*6h00*	*10h05*	*6h25*

Testing	Session 4	2h05	2h00	2h00	2h05

**TABLE II:** TABLE SHOWING THE RESULTS FROM ALL DATASET GROUPS, INCLUDING SUBJECT GROUPING (USER-DEPENDENT CLASSIFICATION (DC) AND USER-INDEPENDENT (IC)), SESSION BLOCKS (BLOCK^1^, BLOCK^2^, AND BLOCK^3^), AND WITHOUT ADAPTATION (CONTROL) OR WITH ADAPTATION. AVG. REPRESENTS THE COMBINED WEIGHTED AVERAGE ACROSS STEADY-STATE (SS) AND TRANSITIONAL (T) STEPS (% [SEM])

		Control		Adaptation	

		DC	IC	DC	IC

Block^1^	SS/T Avg.	3.71 [2.28] 6.39[0.60] 4.09[1.87]	3.12[1.29] 10.67[1.32] 4.19[1.25]	0.58[0.07] 5.97[1.25] 1.34[0.18]	0.67[0.11] 8.88[1.06] 1.83[0.21]

Block^2^	SS/T Avg.	1.44[0.73] 5.34[0.50] 1.99[0.67]	1.83 [0.60] 10.88[1.62] 3.11 [0.68]	0.50[0.12] 4.49[1.03] 1.07 [0.21]	0.61 [0.11] 8.40[0.64] 1.71 [0.17]

Block^3^	SS/T Avg.	0.30[0.07] 5.07 [0.82] 0.97[0.13]	1.59[0.58] 11.09 [2.06] 2.93[0.73]	0.40[0.03] 3.91 [0.98] 0.90[0.12]	0.51 [0.12] 8.24[0.70] 1.61[0.15]

**TABLE III:** TABLE SHOWING THE DISTRIBUTION OF ERROR ACROSS LOCOMOTION MODES (STANDING (ST), LEVEL-GROUND WALKING (LW), STAIR ASCENT (SA), STAIR DESCENT (SD), AND RAMP DESCENT (RD)) FROM ALL DATASET GROUPS, INCLUDING SUBJECT GROUPING (USER-DEPENDENT CLASSIFICATION (DC) AND USER-INDEPENDENT (IC)), SESSION BLOCKS (BLOCK^1^, BLOCK^2^, AND BLOCK^3^), AND WITHOUT ADAPTATION (CONTROL) OR WITH ADAPTATION. VALUES DISPLAYED IN BOLD INDICATE THE ACCURACY OF A CORRECT PREDICTION

Control											

				DC					IC		

		ST	LW	SA	SD	RD	ST	LW	SA	SD	RD

	ST	**0.06**	100.00	99.94	100.00	100.00	**1.79**	100.00	98.41	99.80	100.00
	LW	100.00	**4.83**	95.51	99.87	99.79	100.00	**2.58**	97.85	99.74	99.82
Block^1^	SA	98.76	97.37	**3.87**	100.00	100.00	94.27	89.78	**15.94**	100.00	100.00
	SD	99.66	93.83	100.00	**6.96**	99.55	98.43	97.53	100.00	**4.04**	100.00
	RD	100.00	77.58	98.73	98.41	**25.28**	100.00	75.20	100.00	97.46	**27.34**

	ST	**0.04**	100.00	99.96	100.00	100.00	**1.05**	100.00	98.95	100.00	100.00
	LW	100.00	**0.50**	99.81	99.77	99.92	100.00	**0.81**	99.64	99.81	99.74
Block^2^	SA	99.69	87.77	**12.54**	100.00	100.00	97.21	87.62	**15.17**	100.00	100.00
	SD	99.78	97.42	100.00	**2.92**	99.89	97.98	96.07	99.89	**6.17**	99.89
	RD	100.00	81.08	100.00	98.73	**20.19**	100.00	77.27	100.00	97.14	**25.60**

	ST	**0.06**	100.00	99.94	100.00	100.00	**1.35**	100.00	98.67	99.98	100.00
	LW	100.00	**0.24**	99.90	99.97	99.89	100.00	**1.07**	99.50	99.85	99.58
Block^3^	SA	99.54	98.45	**2.01**	100.00	100.00	97.37	92.57	**10.06**	100.00	100.00
	SD	99.55	98.88	99.89	**1.68**	100.00	98.09	97.31	100.00	**4.60**	100.00
	RD	100.00	87.76	100.00	98.89	**13.35**	100.00	77.90	100.00	97.77	**24.32**

Adaptation											

				DC					IC		

		ST	LW	SA	SD	RD	ST	LW	SA	SD	RD

	ST	**0.34**	100.00	99.68	99.98	100.00	**0.34**	100.00	99.68	99.98	100.00
	LW	100.00	**1.00**	99.68	99.81	99.52	100.00	**1.16**	99.52	99.85	99.47
Block^1^	SA	99.38	96.90	**3.72**	100.00	100.00	97.83	95.05	**7.12**	100.00	100.00
	SD	99.78	98.20	100.00	**2.13**	99.89	99.10	98.20	99.78	**3.25**	99.66
	RD	100.00	91.89	100.00	98.89	**9.22**	100.00	88.08	100.00	99.05	**12.88**

	ST	**0.24**	100.00	99.76	100.00	100.00	**0.30**	100.00	99.70	100.00	100.00
	LW	100.00	**0.58**	99.85	99.90	99.66	100.00	**1.10**	99.58	99.82	99.50
Block^2^	SA	99.54	97.21	**3.25**	100.00	100.00	98.14	97.21	**4.64**	100.00	100.00
	SD	99.78	98.09	100.00	**2.36**	99.78	99.33	98.09	100.00	**3.03**	99.55
	RD	100.00	92.37	100.00	99.21	**8.43**	100.00	87.12	100.00	98.73	**14.15**

	ST	**0.20**	100.00	99.80	100.00	100.00	**0.30**	100.00	99.70	100.00	100.00
	LW	100.00	**0.42**	99.94	99.94	99.71	100.00	**1.15**	99.53	99.82	99.50
Block^3^	SA	99.69	98.45	**1.86**	100.00	100.00	98.14	96.75	**5.11**	100.00	100.00
	SD	99.78	99.10	99.89	**1.23**	100.00	99.33	98.54	100.00	**2.47**	99.66
	RD	100.00	90.78	100.00	99.52	**9.70**	100.00	89.19	100.00	99.05	**11.76**

**TABLE IV:** MIXED MODEL RESULTS FOR FIXED EFFECTS. BLACK BACKGROUND INDICATES SIGNIFICANCE OF *P* < 0.01, GRAY BACKGROUND INDICATES SIGNIFICANCE OF *P* < 0.05, AND WHITE BACKGROUND INDICATES NO SIGNIFICANCE (*P* > 0.05). ‘BLOCK’: BLOCK^1^, BLOCK^2^, BLOCK^3^; ’CLASSIFICATION’: USER-DEPENDENT CLASSIFICATION, USER-INDEPENDENT CLASSIFICATION; ‘ADAPTATION’: CONTROL GROUP, ADAPTATION GROUP

Tests of Fixed Effects		

	F-Value	P-Value
	
Block	3.31	0.048
Classification	3.62	0.065
Adaptation	12.13	0.001
Block*Classification	0.57	0.572
Block* Adaptation	1.90	0.164
Classification*Adaptation	0.22	0.640
Block*Classification*Adaptatio	n 0.35	0.704
